# Optical Nanoparticles for Biomedicine

**DOI:** 10.3390/biomedicines10081892

**Published:** 2022-08-05

**Authors:** José García Solé

**Affiliations:** Nano-Big Group, Department of Materials Science, Faculty of Sciences, Universidad Autónoma de Madrid, 28049 Madrid, Spain; jose.garcia_sole@uam.es

A great variety of particles of different compositions and shapes with typical sizes in the range 1–200 nm have been developed during the first years of this century. These small particles, typically known as nanoparticles, provide a variety of potential applications in biomedicine, such as high-resolution bioimaging and personalized local therapies. Types of nanoparticles used up to now include different organic and inorganic compounds.

Nowadays nanoparticles can behave as intelligent nano-vehicles that can flow throughout the blood flow and, when properly surface-functionalized, localize in specific sites of interest, such as tumors or even specific cells. The so-called optical nanoparticles are those generating any kind of energy (light, heat, or photoacoustic) upon illumination with optical radiation. They have been successfully applied for non-invasive imaging and/or therapeutics.

This Special Issue of *Biomedicines* includes original works related to optical nanoparticles that produce fluorescence (fluorescent nanoparticles). Two works are related to the so-called upconversion nanoparticles (UCNPs). These nanoparticles consist of nanocrystals doped with two kind of trivalent lanthanide ions (Yb^3+^ absorbing ions) and (Er^3+^ or Tm^3+^ emitting ions). Yb^3+^ ions are excited by near-infrared (NIR) light (around 980 nm) and the absorbed energy is transferred to the emitting ions to generate different visible luminescence bands by means of a process known as upconversion energy transfer. Thus, UCNPs transform the NIR-absorbed light into visible fluorescence, so that the autofluorescence and the scattered excitation radiation from biological samples are practically eliminated. Moreover, UCNPs do not display photo-blinking, are very resistant to photo-bleaching and, in principle, display relatively low cytotoxicity. Consequently, UCNPs have been successfully used for a variety of applications, including autofluorescence free fluorescence imaging, sensing, drug delivery and other different therapeutic applications.

In order to make baser UCNPs biocompatible, they must be properly coated with suitable molecules to be water dispersible, i.e., with a proper hydrophilic coating. In addition, although it is generally considered that UCNPs are non-toxic materials, the release of fluorides and lanthanides upon their dissolution may cause cytotoxicity. In this respect, an interesting approach is here performed by J. F. Ferrera et al. [1]. In this work, they successfully synthetized inorganic-organic nanohybrids consisting of NaYF_4_; Yb^3+^, Er^3+^ (UCNPs) coated with different cucurbiturils and investigated their cytotoxicity. The internalization of these nanohybrids in different cell lines was visualized by the visible Er^3+^ ion upconverted luminescence. These nanohybrids, highly stable in aqueous solutions, were demonstrated to be non-cytotoxic to endothelial cells, but displayed a slightly higher cytotoxicity to HeLa and RAW 264.7 cells.

E. Moyano et al. used the blue emitted light of NaYF_4_; Yb^3+^, Tm^3+^ UCNPs (due to Tm^3+^ ions) for light-driven drug release of Ciprofloxacin, an antibiotic that has been commonly used to treat bacterial infections as well as to prevent prostate or lung cancer [2]. All this was driven under NIR excitation light. The emitted blue light induced an efficient delivery of the drug, which cannot be directly used as it is biologically incompatible. Indeed, the strategy used in this work involves a novel methodology; the use of a prodrug (oxime ester of Ciprofloxacin) in which specific bonds are first cleaved by means of the blue light luminescence produced by the UNCPs to finally deliver the active Ciprofloxacin drug.

A variety of optical nanoparticles produce NIR-emitted light under NIR excitation. This is essential for some biomedical applications, particularly for in vivo imaging, as the maximum tissue transparency lies just within the NIR. However, the NIR light emission is usually accompanied by heat generation, and so it is utmost importance to ensure the rational design of nanoparticles for minimizing heat production. In this Special Issue, a review due to K. Okubo et al. is dedicated to investigating the main guidelines to design hybrid nanostructures for minimizing heat generation by using surrounding low vibrational centers or molecules with small chemical polarity [3].

Exosomes are the smallest extracellular vesicles (50–150 nm) and constitute a novel alternative to synthetic nanoparticles for imaging and drug delivery. Exosomes could work as natural bio-targeting nanoparticles promising better accumulation properties compared to synthetic nanoparticles. Fluorescence imaging using these natural nanoparticles can provide accurate (non-invasive) in vivo information on their pharmacokinetic properties and biodistribution.

In this Special Issue, M. I. Gonzalez et al. describe a novel straightforward method for labeling exosomes (isolated from goat’s milk) with two different commercial fluorescent dyes by means of a covalent bond between the ester groups of the fluorophores and the amine groups present in the exosomes [4]. These fluorescent exosomes were successfully used to obtain in vitro (U 87 and B16F10 cancer cells), in vivo and ex vivo (mice) fluorescence imaging. The fluorescence images of these nanoparticles in healthy mice allowed them to evaluate their biodistribution over time. At the cellular level, they demonstrated that their fluorescent exosomes were rapidly accumulated around the cell nuclei in a dose dependent manner.

Nanostructures for multimodal imaging at the molecular level, i.e., that can be visualized by means of different imaging techniques, are another field of increasing activity.

Fernando Oliveira et al. used superparamagnetic iron oxide nanoparticles (SPIONs), conjugated with two types of fluorophores, as an innovative way to evaluate the homing and tracking of hematopoietic stem cells in a bone marrow transplant model in young and old mice [5]. These studies are essential in order to detect the number of transplanted cells reaching the sites of interest in order to properly re-establish bone narrow function.

SPIONs are excellent contrast agents for magnetic resonance imaging (MRI), while the attached fluorophores (one absorbing and emitting in the NIR region and the other, Rhodamine-B, which absorbs and emits in the visible region) provide the additional possibility of using fluorescence imaging techniques. Thus, these nanoparticles are excellent candidates for multimodal imaging, and more importantly, for imaging at the molecular level. They performed systematic in vitro studies by means of fluorescence imaging, taking advantage of Rhodamine-B luminescence to visualize the internalization in bone marrow mononuclear cells. Then they quantified the internalized SPION nanoparticles in these cells in correlation to the NIR fluorescence and the intrinsic magnetic resonance contrast of iron oxide (MRI imaging). These experiments were then applied to in vivo and ex vivo studies to evaluate aging effects in the homing and tracking of SPIONs-labelled hematopoietic stems cells during a bone narrow transplant, using old and young mice. In addition, bioluminescence was used to follow the long-term grafting of these cells into the bone marrow.

In summary, the works included in this special issue evidence the great activity in the use of fluorescent nanoparticles for different biomedical applications. It will incentivize new scientist to use novel designs of optical nanostructures for future personalized biomedicines.
